# Gender-specific analysis for the association between trunk muscle mass and spinal pathologies

**DOI:** 10.1038/s41598-021-87334-4

**Published:** 2021-04-09

**Authors:** Yusuke Hori, Masatoshi Hoshino, Kazuhide Inage, Masayuki Miyagi, Shinji Takahashi, Shoichiro Ohyama, Akinobu Suzuki, Tadao Tsujio, Hidetomi Terai, Sho Dohzono, Ryuichi Sasaoka, Hiromitsu Toyoda, Minori Kato, Akira Matsumura, Takashi Namikawa, Masahiko Seki, Kentaro Yamada, Hasibullah Habibi, Hamidullah Salimi, Masaomi Yamashita, Tomonori Yamauchi, Takeo Furuya, Sumihisa Orita, Satoshi Maki, Yasuhiro Shiga, Masahiro Inoue, Gen Inoue, Hisako Fujimaki, Kosuke Murata, Ayumu Kawakubo, Daijiro Kabata, Ayumi Shintani, Seiji Ohtori, Masashi Takaso, Hiroaki Nakamura

**Affiliations:** 1 Department of Orthopaedic Surgery, Osaka City University Graduate School of Medicine, 1-4-3 Asahimachi, Abeno-ku, Osaka, 545-8585 Japan; 2 Department of Orthopaedic Surgery, Graduate School of Medicine, Chiba University, Chiba, Japan; 3 Department of Orthopaedic Surgery, School of Medicine, Kitasato University, Sagamihara, Kanagawa Japan; 4 Department of Orthopaedic Surgery, Shiraniwa Hospital, Nara, Japan; 5 Department of Orthopaedic Surgery, Yodogawa Christian Hospital, Osaka, Japan; 6 Department of Orthopaedic Surgery, Osaka City General Hospital, Osaka, Japan; 7 Department of Orthopedic Surgery, JCHO Funabashi Central Hospital, Chiba, Japan; 8 Department of Orthopedic Surgery, Asahi General Hospital, Chiba, Japan; 9 Department of Medical Statistics, Osaka City University Graduate School of Medicine, Osaka, Japan; 10 Chiba University Center for Frontier Medical Engineering, Chiba, Japan

**Keywords:** Quality of life, Muscle, Disability, Chronic pain

## Abstract

We investigated the relationship between trunk muscle mass and spinal pathologies by gender. This multicenter cross-sectional study included patients aged ≥ 30 years who visited a spinal outpatient clinic. Trunk and appendicular muscle mass were measured using bioelectrical impedance analysis. The Oswestry Disability Index (ODI), visual analog scale (VAS) score for low back pain, sagittal vertical axis (SVA), and EuroQol 5 Dimension (EQ5D) score were investigated to evaluate spinal pathology. The association between trunk muscle mass and these parameters was analyzed by gender using a non-linear regression model adjusted for patients’ demographics. We investigated the association between age and trunk muscle mass. We included 781 men and 957 women. Trunk muscle mass differed significantly between men and women, although it decreased with age after age 70 in both genders. Lower trunk muscle mass was significantly associated with ODI, SVA, and EQ5D score deterioration in both genders; its association with VAS was significant only in men. Most parameters deteriorated when trunk muscle mass was < 26 kg in men and < 19 kg in women. Lower trunk muscle mass was associated with lumbar disability, spinal imbalance, and poor quality of life in both genders, with significant difference in muscle mass.

## Introduction

Japan is the world’s leading “super-aged” society^1^, making extending healthy life expectancy an urgent issue. Overcoming musculoskeletal diseases is an important issue in extending a healthy lifespan. Low back pain, a musculoskeletal disease, is now the most frequent cause of disability worldwide^2^, and is increasing in frequency as the population ages. Age-related spinal deformities caused by lumbar degenerative kyphosis and osteoporotic vertebral fractures also cause stiff back pain and gait disturbance, severely disrupting the daily lives of the elderly^3^. Therefore, elucidating the pathogenesis of and preventing low back pain and spinal deformity are crucial to achieve a society where the elderly live a healthy and active life.

The trunk muscles are critical supportive elements of the spine and contribute significantly to various spinal pathologies^4,5^. However, these muscles decrease with age, a condition termed as sarcopenia. Although its pathomechanism has not yet been fully understood, aging appears to result in an imbalance between muscle protein anabolic and catabolic pathways^6^. Trunk muscle mass has also been proven to decline with age^7^, requiring further exploration of its pathogenesis.

Gender is a significant factor in defining muscle mass, as muscle mass has been reported to be greatly different between men and women^8^. There are also gender differences in trunk muscle mass; trunk muscle mass is larger in men than in women^9^. The occurrence of muscle atrophy also differs between men and women, and may be influenced by the composition of muscle fibers^10^. Men have more type 2a muscle fibers, while women have more type 1 muscle fibers, which makes women more susceptible to disuse atrophy^11^. The structural changes at the neuromuscular junctions associated with aging are also considered to be a crucial factor in causing muscle atrophy^12^. These junctions should also be considered in relation to gender, since there is evidence that men and women have different neuromuscular adaptations to unloading^13^. Furthermore, a current study reviewed that men and women clearly differ in many aspects of muscle health and physiology, including anabolic and catabolic pathways, hormonal interactions, and mitochondrial content and function^10^. Gender differences have been reported to exist not only in muscles but also in spinal pathologies. The prevalence of low back pain was reported to be higher in women than in men, and as high as 1.28-fold among the elderly (≥ 50 years old)^14^. There are also gender differences in spinal alignment, especially in pelvic incidence and pelvic tilt, which have been reported to be larger in women^15^. These underlying gender differences in muscles and spinal pathologies suggest the importance of a gender-specific analysis in investigating the association between trunk muscles and spinal pathologies.

A previous study^7^ clarified the clinical importance of trunk muscle mass in association with disability, low back pain, spinal deformity, and poor quality of life (QOL), which started to deteriorate with trunk muscle mass approximately < 23 kg. However, optimal trunk muscle mass depends on the individual and can be greatly influenced by gender. Although gender was considered in the analysis, further detailed evaluation by gender was required. In this study, we aimed to evaluate the association between trunk muscle mass and these spinal pathologies by gender, which is a critical determinant factor of muscle mass, along with age-related decline in trunk and appendicular muscle mass.

## Results

This study included 781 men (mean age 69.3 ± 11.4 years) and 957 women (mean age 70.9 ± 10.5 years). Table 1 shows the demographic data of the subjects. Men had significantly more trunk muscle mass (26.1 ± 3.4 kg vs 19.1 ± 2.0 kg, *p* < 0.001) and appendicular skeletal muscle mass (22.4 ± 4.0 kg vs 14.5 ± 2.4 kg, *p* < 0.001) than women.

**Table 1:** Demographics of subjects.

Characteristic	n (%) or mean (SD)		*p*^a^ value
	Men (n = 781)	Women (n = 957)	
Age, years	69.3 (11.4)	70.9 (10.5)	0.004
BMI, kg/m^2^	24.1 (3.6)	22.7 (3.9)	< 0.001
Trunk muscle mass, kg	26.1 (3.4)	19.1 (2.0)	< 0.001
Appendicular skeletal muscle mass, kg	22.4 (4.0)	14.5 (2.4)	< 0.001
ODI	20.3 (16.8)	24.3 (17.8)	< 0.001
VAS scale of low back pain, mm	31.0 (28.0)	33.6 (28.3)	0.068
SVA, mm	37.4 (38.2)	40.2 (49.3)	0.211
EQ5D	0.79 (0.19)	0.76 (0.19)	0.025
Charlson comorbidity index	1.2 (3.0)	0.9 (2.6)	0.033
**Main disorders**			0.005
Lumbar spinal stenosis	387 (49.5)	346 (36.1)	
Cervical spinal stenosis	109 (14.0)	85 (8.8)	
Disc herniation	67 (8.6)	62 (6.5)	
Vertebral fractures	19 (2.4)	99 (10.3)	
Osteoporosis	10 (1.3)	161 (16.8)	
Deformity	8 (1.1)	46 (4.9)	
Spondylosis	52 (6.7)	57 (5.9)	
Spinal tumor	42 (5.4)	34 (3.6)	
Ossifications	25 (3.3)	13 (1.4)	
Others	39 (5.0)	40 (4.2)	
Not available	21 (2.7)	14 (1.5)	
History of lumbar surgery	301 (38.5)	229 (23.9)	< 0.001

*BMI* body mass index, *EQ5D* EuroQol 5 Dimension, *ODI* Oswestry disability index, *SVA* sagittal vertical axis, *VAS* visual analog scale.

^a^The chi-squared test was used for categorical variables, and the *t* test was used for continuous variables.

**Figure 1:**
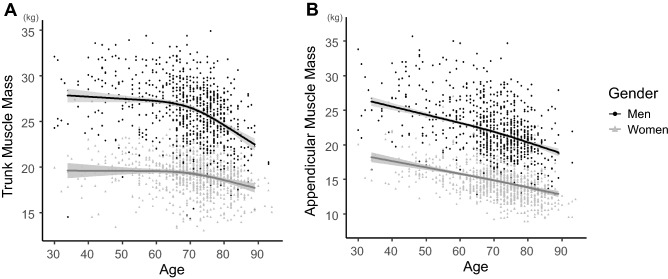
(**A**) Association between age and trunk muscle mass adjusted for body mass index (BMI). Trunk muscle mass declined with age in both men and women (*p* < 0.001, *p* for non-linear < 0.001). (**B**) Association between age and appendicular skeletal muscle mass adjusted for BMI. Appendicular skeletal muscle mass declined with age in both genders (men: *p* < 0.001, *p* for non-linear = 0.243; women: *p* < 0.001, *p* for non-linear = 0.721). Gray zone indicates 95% confidence intervals.

Trunk muscle mass had a negative association with age in both men and women (*p* < 0.001). Men maintained their trunk muscle mass of ≥ 26 kg until their 60 s, which then declined rapidly in their 70 s. The average slopes (βs) of the non-linear curve within a particular range were − 0.03 for 30–70 years of age and − 0.20 for 70–90 years of age. Meanwhile, women preserved approximately ≥ 19 kg of their trunk muscle mass until their 60 s (β = − 0.01), which also began to decline in their 70 s (β = − 0.09) (Fig. 1A). Appendicular skeletal muscle mass was also negatively associated with age, with consistent decreases in both men and women (*p* < 0.001) (Fig. 1B).

**Figure 2:**
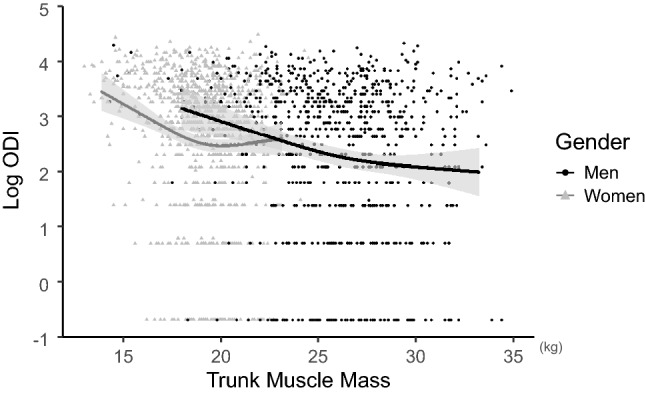
Association between trunk muscle mass and log of the Oswestry Disability Index (ODI) adjusted for patients’ demographics. Lower trunk muscle mass significantly associated with deterioration of log ODI in both genders (men: *p* < 0.001, *p* for non-linear = 0.127; women: *p* < 0.001, *p* for non-linear = 0.002). Gray zone indicates 95% confidence intervals.

**Figure 3:**
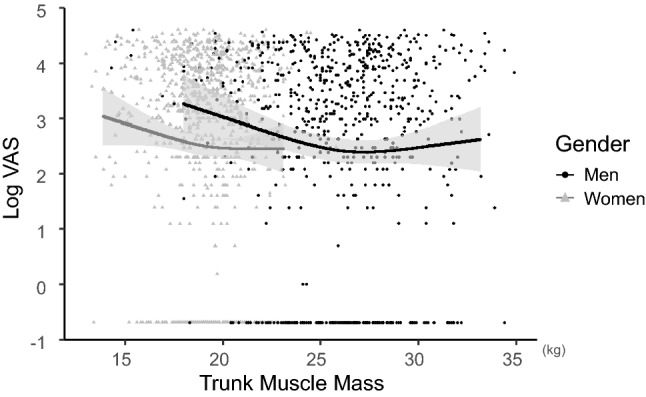
Association between trunk muscle mass and log of visual analog scale (VAS) score for low back pain adjusted for patients’ demographics. Lower trunk muscle mass significantly associated with increased log of the VAS score only for men (men: *p* = 0.015, *p* for non-linear = 0.038; women: *p* = 0.094, *p* for non-linear = 0.237). Gray zone indicates 95% confidence intervals.

**Figure 4:**
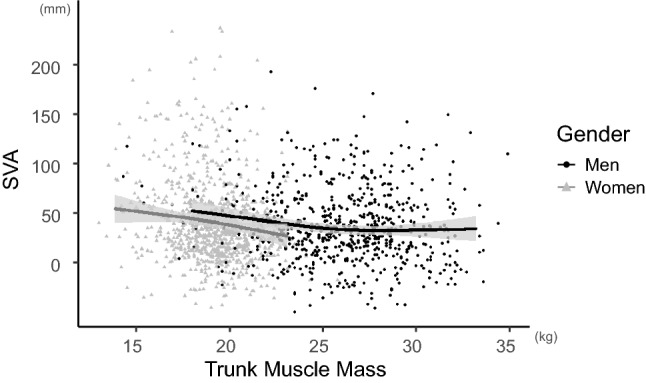
Association between trunk muscle mass and sagittal vertical axis (SVA) adjusted for patients’ demographics. Lower trunk muscle mass significantly associated with increased SVA in both genders (men: *p* = 0.008, *p* for non-linear = 0.081; women: *p* = 0.006, *p* for non-linear = 0.865). Gray zone indicates 95% confidence intervals.

**Figure 5:**
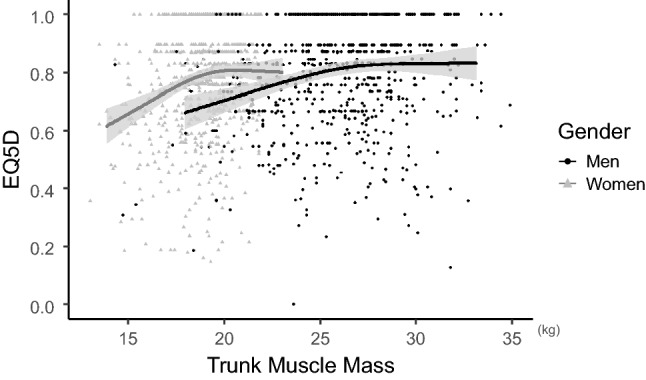
Association between trunk muscle mass and EuroQoL 5 dimension (EQ5D) adjusted for patients’ demographics. Lower trunk muscle mass significantly associated with poor EQ5D in both genders (men: *p* < 0.001, *p* for non-linear = 0.014; women: *p* < 0.001, *p* for non-linear = 0.003). Gray zone indicates 95% confidence intervals.

Trunk muscle mass was negatively associated with the Oswestry Disability Index (ODI) in both genders (*p* < 0.001) when adjusted for age, body mass index (BMI), Charlson Comorbidity Index (CCI), appendicular skeleecatal muscle mass, and history of lumbar surgery. Deterioration of the ODI accelerated in men when the trunk muscle mass was approximately < 26 kg, while it began in women with trunk muscle mass of approximately < 19 kg (Fig. 2). The βs of the non-linear curve within a particular range were − 0.04 for trunk muscle mass > 26 kg (< 33 kg) and − 0.11 for trunk muscle mass < 26 kg (> 18 kg) in men. In women, β was 0.01 when trunk muscle mass was > 19 kg (< 23 kg) and − 0.18 when trunk muscle mass was < 19 kg (> 14 kg). Trunk muscle mass was also negatively associated with the visual analog scale (VAS) score, with a significant difference in men (*p* = 0.015). In men, log VAS was almost constant when trunk muscle mass was approximately > 26 kg (β = 0.03), while it increased with decreasing trunk muscle mass when trunk muscle mass was approximately < 26 kg (β = − 0.10). In women, there was no significant difference between trunk muscle mass and log VAS (*p* = 0.094) (Fig. 3). The relationship between trunk muscle mass and sagittal vertical axis (SVA) was investigated in 688 men (mean age 69.6 ± 11.2 years) and 857 women (mean age 71.1 ± 10.2 years) who were available for standing whole-spine radiographs. Trunk muscle mass had a significant negative correlation with SVA in both genders (*p* = 0.008 for men; *p* = 0.006 for women) when adjusted for confounding factors. In men, SVA was almost constant when trunk muscle mass was approximately > 26 kg (β = 0.07), whereas SVA increased as trunk muscle mass decreased when trunk muscle mass was approximately < 26 kg (β = − 2.29). In women, SVA presented consistent deterioration with the decrease of trunk muscle mass (β = − 2.51 when trunk muscle mass < 19 kg; − 3.47 when trunk muscle mass > 19 kg) (Fig. 4). Trunk muscle mass was positively correlated with the EuroQol 5 Dimension (EQ5D) score in both genders when adjusted for confounding factors (*p* < 0.001). Subjects with a high trunk muscle mass presented a high EQ5D score, which deteriorated when trunk muscle mass was approximately < 26 kg in men (β = 0.019 when trunk muscle mass < 26 kg; 0.003 when trunk muscle mass > 26 kg) and approximately < 19 kg in women (β = 0.035 when trunk muscle mass < 19 kg; 0.007 when trunk muscle mass > 19 kg) (Fig. 5).

Results of the sub-analysis are shown in Supplementary Figs. S1–S4. When patients were divided into quartiles based on ODI, trunk muscle mass in men was significantly lower in quartiles 3 (ODI: 20–34) and 4 (34–88.9) and in quartile 4 in women (0–7.62) (Supplementary Fig. S1). When patients were divided into quartiles by the VAS score, trunk muscle mass was significantly lower in quartile 4 (VAS: 54–100) than in quartile 1 (0–7) for both men and women (Supplementary Fig. S2). When patients were divided into three categories by SVA, trunk muscle mass was significantly lower in category 2 (SVA: 40–95 mm) in men and in categories 2 and 3 (SVA > 95 mm) in women (SVA < 40 mm) (Supplementary Fig. S3). When patients were divided into quartiles by the EQ5D, trunk muscle mass was significantly lower in quartiles 1 (0.001–0.065), 2 (0.665–0.82), and 3 (EQ5D: 0.82–0.895) in men and in quartile 1 in women (0.895–1) (Supplementary Fig. S4).

To eliminate confounding between clinical scores, multivariate analysis was performed with decreased trunk muscle mass as the objective variable and clinical score as the explanatory variable. Since ODI and EQ5D are strongly correlated (r = − 0.801 for men; − 0.803 for women), the EQ5D was excluded from the analysis to avoid collinearity. ODI alone was significantly associated with trunk muscle mass in men (Table 2). In women, both ODI and SVA were significantly associated with trunk muscle mass (Table 3).

**Table 2:** Association between trunk muscle mass and clinical parameters for men.

	β	95% CI	*p* value
Age, years	− 0.079	− 0.100 to 0.058	< 0.001
BMI, kg/m^2^	0.275	0.208–0.342	< 0.001
ODI	− 0.039	− 0.056 to 0.021	< 0.001
VAS scale of low back pain, mm	0.006	− 0.004 to 0.017	0.242
SVA, mm	− 0.005	− 0.011 to 0.001	0.105

*BMI* body mass index, *ODI* Oswestry disability index, *SVA* sagittal vertical axis, *VAS* visual analog scale.

**Table 3:** Association between trunk muscle mass and clinical parameters for women.

	β	95% CI	*p* value
Age, years	− 0.029	− 0.043 to 0.015	< 0.001
BMI, kg/m^2^	0.037	0.002–0.072	0.040
ODI	− 0.018	− 0.027 to 0.008	< 0.001
VAS scale of low back pain, mm	0.002	− 0.004 to 0.007	0.607
SVA, mm	− 0.004	− 0.007 to 0.001	0.011

*BMI* body mass index, *ODI* Oswestry disability index, *SVA* sagittal vertical axis, *VAS* visual analog scale.

## Discussion

Muscle mass including trunk muscles is known to differ significantly between men and women. Johannesdottir et al.^9^ quantitatively investigated the computed tomography scans of 250 community-based men and women aged 40–90 years, and identified that trunk muscles in men were 20–67% larger than in women. They also reported that trunk muscle size decreases with age by ~ 8% per decade in both men and women. This study revealed that trunk muscle mass as well as appendicular muscle mass differed significantly between men and women, with men averaging ~ 7 kg (37%) more muscle mass than women. Aging and muscle mass loss was significantly related among both cohorts of men and women. Appendicular muscle mass had a linear association with age, whereas trunk muscle mass had a nonlinear association with age in both genders. Appendicular muscle mass declined at a constant rate with age in both men and women. Although trunk muscle mass was generally maintained at a minimum of 26 kg for men and 19 kg for women until the age of 60 years, it declines rapidly after 70 years of age. The age-related decreases in muscle mass after 70 years of age were 2.0 kg (7.7%) in men and 0.9 kg (4.7%) in women per decade. The difference between appendicular and trunk muscle mass may be accounted for by the fact that the trunk muscles, which contain more type 1 fibers, are less susceptible to the effects of aging^16^. Regardless of the amount, trunk muscle mass declines rapidly in both men and women as they get older, requiring that both elderly men and women should make an effort to maintain their trunk muscles.

As pain mechanisms may differ between genders^17^, possible causes of low back pain may also differ between genders. This study showed that the VAS for low back pain was negatively associated with trunk muscle mass, which was statistically significant only in men. The association was not significant in women, possibly due to a multiplicity of factors causing back pain, one of which is vertebral fractures. This study showed a higher prevalence of vertebral fractures in women than in men (8.3% in men, 18.7% in women). As greater effects of metabolic syndrome and obesity on back pain have been reported in women^18^, causes other than trunk muscle mass loss might need to be considered to improve back pain. On the other hand, decreased trunk muscle mass was significantly associated with deteriorated ODI in both men and women. Addittionally, the ODI was significantly associated with trunk muscle mass independent of age, BMI, or SVA in both men and women, whereas it was not significantly associated with low back pain or VAS, suggesting that loss of trunk muscle mass might contribute to disability rather than the pain itself. The optimal trunk muscle mass varies among individuals, and one of the critical determinants is gender. The ODI decreased with lower trunk muscle mass, especially in patients with trunk muscle mass of < 19 kg for women and < 26 kg for men. These results support the possibility that optimal trunk muscle mass may differ significantly between genders and, furthermore, that maintaining trunk muscle mass may improve the disability associated with low back pain.

Deterioration of spinal alignment with aging is well-documented, which can develop differently in men and women. Oe et al.^19^ reported in a 4-year longitudinal study that spinal deformity starts with deterioration in pelvic alignment, with earlier deterioration in women than in men. In the present study, trunk muscle mass decline was significantly associated with deterioration of SVA in both men and women. Deterioration of SVA occurs in men with < 26 kg of trunk muscle mass, whereas it increased linearly with decreasing trunk muscle mass in women. Moreover, multivariate analysis revealed that SVA was significantly associated with trunk muscle mass independent of ODI and VAS in women, while it was not an independently associated factor in men. These results suggest that lower trunk muscle mass in men may be associated with increased SVA deterioration, causing low back pain and disability, whereas lower trunk muscle mass may be directly associated with increased SVA deterioration in women. Women with reduced trunk muscle mass may quickly develop spinal imbalance, which may be related to the occurrence of vertebral fractures. Given that stronger back muscles inhibit the incidence of vertebral fractures^20^, it is conceivable that lower trunk muscle mass might affect spinal sagittal balance via vertebral fractures. In addition, the incidence of vertebral fracture increases fat infiltration in paravertebral muscles^21^. Further investigations including longitudinal studies are needed to elucidate the causal relationship between decreases in trunk muscle mass, spinal kyphosis, and vertebral fracture.

In this study, QOL was also affected by a decrease in trunk muscle mass in both men and women. The reported mean EQ5D score is 0.84 in Japanese men and 0.81 in Japanese women^22^. Subjects with a high trunk muscle mass exhibited nearly average EQ5D score, which declined with decreases in trunk muscle mass from < 26 kg for men and < 19 kg for women. As this study involved patients with spinal disease, it is conceivable that lower trunk muscle mass may contribute to spinal pathology, leading to poor QOL. In fact, the EQ5D score was strongly correlated with the ODI in this study, indicating that disability due to low back pain greatly affected QOL in the patients. Although it is uncertain whether our results are applicable to the general population, considering the association of sarcopenia with poor QOL, maintenance of trunk muscle mass is also important for a good QOL.

This study has several limitations. First, because this study was a cross-sectional study, we could not elucidate the causal relationship between trunk muscle mass and spinal pathology. Whether muscle mass loss comes first or deterioration of spinal pathology is still controversial. Currently, whether low back pain and spinal sagittal imbalance occur because of a decrease in trunk muscle mass or whether trunk muscle mass decreases as a result of low back pain or spinal sagittal imbalance is unknown. Second, we did not limit the type and cause of low back pain to chronic fatigue pain, which would closely relate to trunk muscle mass. Although patients with acute vertebral fractures were excluded, various types of back pain, including discogenic or facet pain, were included, which were possibly reflected in the VAS scores, thereby accounting for its large confidence interval. However, we could prove that the ODI, an indicator of disability due to back pain, as well as the SVA and EQ5D score were significantly associated with trunk muscle mass loss in both men and women. Third, the subjects were patients with spinal disease, and it was uncertain whether these results could be applicable to the general population. In a survey of the general population in Japan consisting of subjects in their 20–70 s, the average ODI values were reported to be 8.8 for men and 8.7 for women, and when limited to those in their 70 s, the values were 10.54 for men and 14.32 for women^23^. Another study of the Japanese general population consisting of subjects older than 50 years of age indicated that the mean SVA was 17 mm for men and 19 mm for women^24^. The mean ODI and SVA of the subjects in this study were higher than those of the general population, indicating that this study might have included participants with more disabilities and kyphosis than the general population. However, given its prevalence, our study subject may be reasonable for the purpose of investigating the association of trunk muscle mass with lumbar disability and spinal deformity. Further investigation on the association between trunk muscle mass and spinal pathology in the general population is needed. Fourth, the ages of the subjects were not evenly distributed. Since this study involved spinal outpatients, the number of younger subjects was relatively small, which may have affected the relationship between age and trunk muscle mass. Finaly, our study only included Japanese subjects. Since muscle mass, prevalence of low back pain, and spinal alignment may vary by race, it is unclear whether the results of this study can be adapted to all races. Validation in other races is necessary to prove the relevance of the results of this study.

In conclusion, lower trunk muscle mass was significantly associated with deterioration of the ODI, SVA, and EQ5D score in both genders, while the association of VAS with low back pain was significant only in men. As trunk muscle mass varies greatly between men and women, these parameters tended to deteriorate when trunk muscle mass was approximately < 26 kg in men and approximately < 19 kg in women. As the decline in trunk muscle mass accelerates for both men and women in their 70 s, it is essential to maintain trunk muscle mass, which might prevent spinal disability and deformity, thereby leading to an extended healthy life expectancy.

## Methods

All study participants provided informed consent. This study was approved by the local ethics committee of the Faculty of Medicine, Osaka City University (Approval No. 3806). All methods were performed in accordance with the Declaration of Helsinki and the Ethical Guidelines for Medical and Health Research Involving Human Subjects in Japan.

### Subjects

This multicenter cross-sectional study comprised of 10 hospitals in three different regions. Subjects were patients who visited the spinal outpatient clinic of one of the 10 hospitals. Bioimpedance analysis (BIA) data were collected from June 2017 to March 2018 for patients who agreed to undergo muscle mass measurement. The inclusion criteria were as follows: (1) over 30 years of age; (2) having some spinal disease (including simple back pain or neck pain) or osteoporosis; (3) able to maintain self-standing. The exclusion criteria were as follows: (1) indwelling a pacemaker; (2) a history of spinal fusion surgery; (3) a history of arthroplasty surgery; (4) having metal in the body, such as surgical materials for fractures; (5) diagnosed with an acute vertebral fracture; and (6) severe dementia.

### Measurement of muscle mass

Whole-body and segmental muscle mass was measured using BIA with a multi-frequency body composition analyzer (MC-780A and MC-980A, Tanita Co., Tokyo, Japan). Subjects were required to stand on the balance scale with bare feet and hold the handles during measurements, which provided whole-body and segmental muscle mass. Muscle mass obtained using BIA has been reported as reliable with a high correlation to dual energy X-ray absorptiometry^25^. Appendicular skeletal muscle mass was calculated as the sum of the skeletal muscle masses of the arms and legs. Trunk muscle mass was calculated by subtracting the appendicular skeletal muscle mass from the whole-body muscle mass. BMI was calculated by dividing the body weight by the square of the height at the time of the measurement.

### Questionnaires

A questionnaire was administered to subjects to determine the impact of back pain on their daily lives and QOL, and to evaluate their comorbidities. The disability related to low back pain was assessed with the ODI, a validated instrument that has been used in several spine patient populations^26^. The ODI questionnaire is composed of ten six-item questionnaires. In addition to pain intensity, it assesses the disabling effects of pain on typical activities of daily living such as personal care, lifting, walking, sitting, standing, sleeping, sex life, social life, and traveling. Each item has a score from 0 to 5, and the sum of the scores of the 10 item is expressed as a percentage of the maximum score, which varies from 0 (no disability) to 100 (maximum disability). The degree of low back pain in the last week was evaluated using the VAS, which represents the intensity dimension of pain by a 100 mm line with two anchors of “no pain” and “worst pain I ever felt”^27^. The 5-level classification system of the EQ5D was used to evaluate QOL, which was the most widely used generic patient-reported outcome questionnaire^28^. The EQ5D comprises of five dimensions: mobility, self-care, usual activities, pain/discomfort and anxiety/depression. Each dimension has 5 levels: no problems, slight problems, moderate problems, severe problems and extreme problems. A summary index with a maximum score of 1 can be derived from these five dimensions by conversion with a table of scores. Comorbidities of subjects were evaluated and categorized based on the CCI^29^, which has been extensively used in clinical research to address the confounding influence of comorbidities. Additionally, history of spinal surgery was collected from medical records and outpatient physicians.

### Radiographic assessment

The SVA was measured with the standing whole-spine radiograph in the lateral view as an assessment of spinal sagittal balance.

### Statistical analysis

For the comparison of baseline clinical and demographical data between male and female subjects, the Chi-squared test and Student’s *t* test were used for categorical and continuous variables, respectively. In order to assess the associations between the trunk muscle mass and some clinical factors (spinal disorders based on the ODI, VAS score, SVA, and EQ5D score) on each gender, we performed multivariable and non-linear regression analyses using subset data consisting of each gender. These models were adjusted for age, BMI, appendicular muscle mass, CCI, and history of lumbar surgery. We also investigated the association between trunk and appendicular muscle mass for each gender using multivariable non-linear regression models adjusted for BMI among each gender dataset. Nonlinearity of the associations between the objective variable and trunk muscle mass was assessed by restricted-cubic-splines in the regression models. The ODI and VAS score were transformed with natural logarithm, as their residuals were not normally distributed. We conducted a sub-analysis by categorizing patients into quartiles based on clinical scores, and compared trunk muscle mass across quartiles for each gender. For SVA, the patients were divided into three groups according to the SRS-Schwab classification (SVA < 40 mm; 40–95 mm; > 95 mm)^30^. An age- and BMI-adjusted multiple linear regression analysis was used to compare trunk muscle mass. Furthermore, to eliminate confounding between clinical scores, these regression analyses were performed for each gender, with trunk muscle mass as the objective variable and clinical scores as the explanatory variables. If the clinical scores were strongly correlated with each other (|r|> 0.8), one of them was excluded from the analysis to remove collinearity. Statistical tests were conducted with two-sided significance level of 5%. All analyses were performed using R (http://www.r-project.org).

### Ethics approval

This study was approved by the local ethics committee of the Faculty of Medicine, Osaka City University (No. 3806).

## Supplementary Information

Supplementary Figures
