# Supplementary material for: Gender-specific analysis for the association between trunk muscle mass and spinal pathologies
Source: Sci Rep. 2021 Apr 9;11:7816. doi: 10.1038/s41598-021-87334-4 (PMC8035169; doi:10.1038/s41598-021-87334-4)
Supplement: Supplementary file 1 — Supplementary Figures [file 41598_2021_87334_MOESM1_ESM.pdf]

Gender-specific analysis for the association between trunk muscle mass and spinal pathologies

Yusuke Hori, MD[1]; Masatoshi Hoshino, MD, PhD[1]*; Kazuhide Inage, MD, PhD[2]; Masayuki Miyagi, MD, PhD[3]; Shinji Takahashi, MD, PhD[1]; Shoichiro Ohyama, MD, PhD[1]; Akinobu Suzuki, MD, PhD[1]; Tadao Tsujio, MD, PhD[4]; Hidetomi Terai, MD, PhD[1]; Sho Dohzono, MD, PhD[5]; Ryuichi Sasaoka, MD, PhD[5]; Hiromitsu Toyoda, MD, PhD[1]; Minori Kato, MD, PhD[6]; Akira Matsumura, MD, PhD[6]; Takashi Namikawa, MD, PhD[6]; Masahiko Seki, MD, PhD[4]; Kentaro Yamada, MD, PhD[1]; Hasibullah Habibi, MD[1]; Hamidullah Salimi, MD[1]; Masaomi Yamashita, MD, PhD[7]; Tomonori Yamauchi, MD, PhD[8]; Takeo Furuya, MD, PhD[2]; Sumihisa Orita, MD, PhD[2, 10]; Satoshi Maki, MD, PhD[2]; Yasuhiro Shiga, MD, PhD[2]; Masahiro Inoue, MD, PhD[2]; Gen Inoue, MD, PhD[3]; Hisako Fujimaki, MD, PhD[3]; Kosuke Murata, MD[3]; Ayumu Kawakubo, MD[3]; Daijiro Kabata, MPH[9]; Ayumi Shintani, PhD, MPH[9]; Seiji Ohtori, MD, PhD[2]; Masashi Takaso, MD, PhD[3]; and Hiroaki Nakamura MD, PhD[1]

[1]Department of Orthopaedic Surgery, Osaka City University Graduate School of Medicine, Osaka, Japan
[2]Department of Orthopaedic Surgery, Graduate school of Medicine, Chiba University, Chiba, Japan
[3]Department of Orthopaedic Surgery, Kitasato University, School of Medicine, Kanagawa, Japan
[4]Department of Orthopaedic Surgery, Shiraniwa Hospital, Nara, Japan
[5]Department of Orthopaedic Surgery, Yodogawa Christian Hospital, Osaka, Japan
[6]Department of Orthopaedic Surgery, Osaka City General Hospital, Osaka, Japan
[7]Department of Orthopedic Surgery, JCHO Funabashi Central Hospital, Chiba, Japan
[8]Department of Orthopedic Surgery, Asahi General Hospital, Chiba, Japan
[9]Department of Medical Statistics, Osaka City University Graduate School of Medicine, Osaka, Japan
[10]Chiba University Center for Frontier Medical Engineering, Chiba, Japan

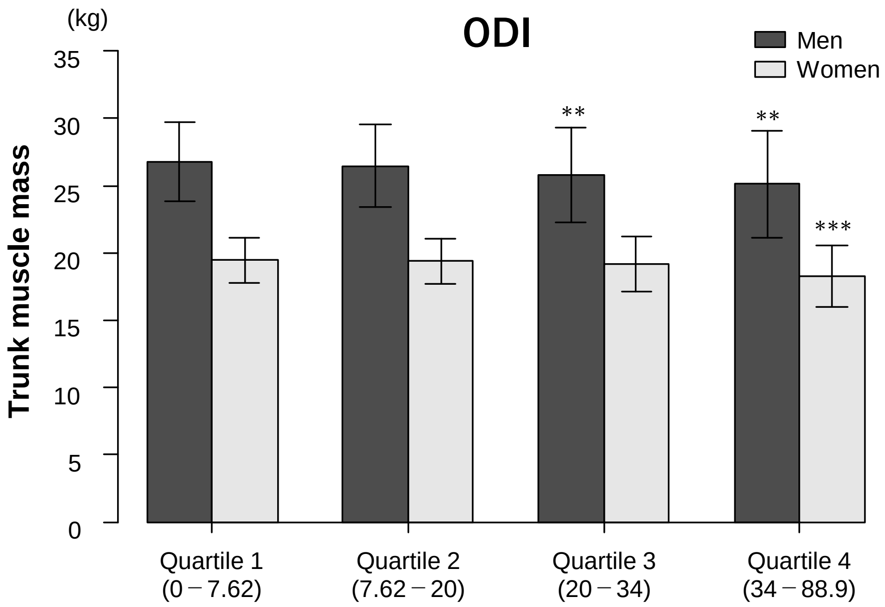

Supplementary Figure S1

Comparison of trunk muscle mass across quartiles of the Oswestry Disability Index (ODI) adjusted for age and body mass index.

*p < 0.05; **p < 0.01; ***p < 0.001 compared with quartile 1.

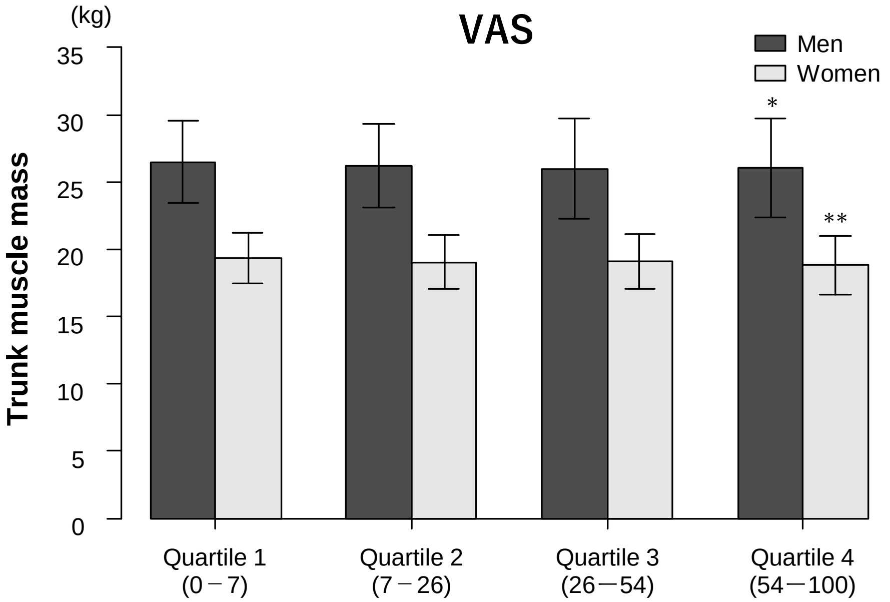

Supplementary Figure S2

Comparison of trunk muscle mass across quartiles of visual analog scale (VAS) score for low back pain adjusted for age and body mass index.

*p < 0.05; **p < 0.01; ***p < 0.001 compared with quartile 1.

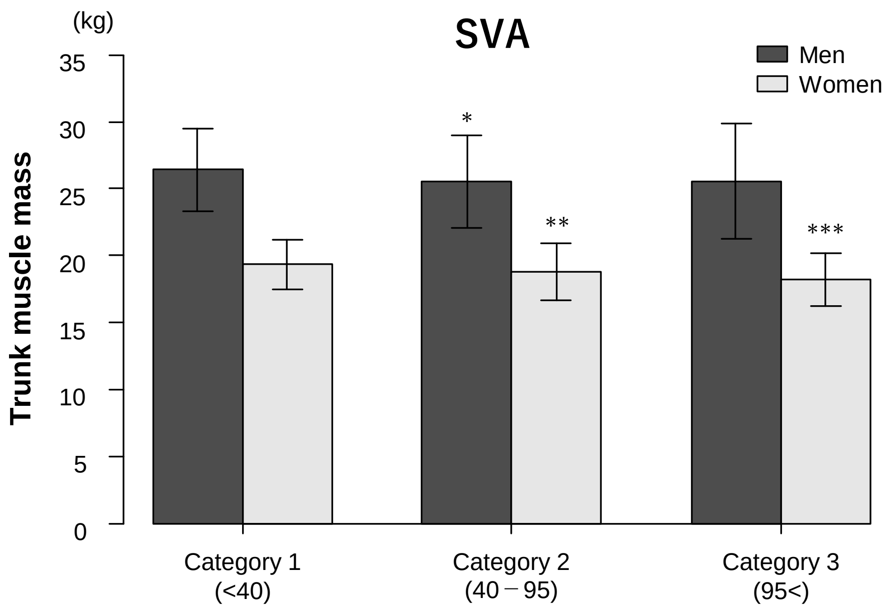

Supplementary Figure S3

Comparison of trunk muscle mass across categories of sagittal vertical axis (SVA) adjusted for age and body mass index.

*p < 0.05; **p < 0.01; ***p < 0.001 compared with category 1.

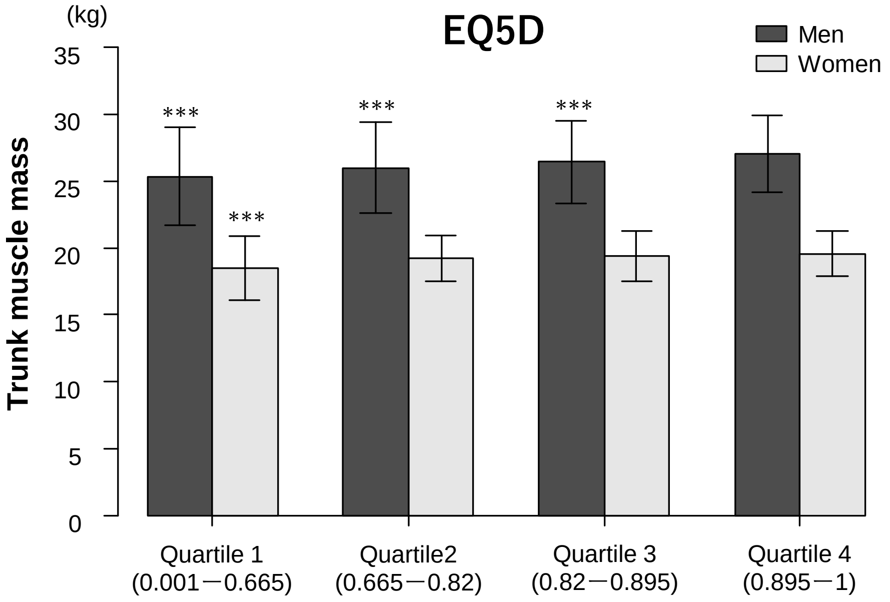

Supplementary Figure S4

Comparison of trunk muscle mass across quartiles of EuroQoL 5 dimension (EQ5D) adjusted for age and body mass index.

*p < 0.05; **p < 0.01; ***p < 0.001 compared with quartile 4.
